# COT-TT vaccine attenuates induction and expression of cocaine-induced behavioral sensitization in rats: a dose-response study

**DOI:** 10.3389/fpsyt.2025.1548585

**Published:** 2025-06-03

**Authors:** Sasana Barbosa-Mendez, Alberto Salazar-Juárez

**Affiliations:** Laboratorio de Neurofarmacología Conductual, Microcirugía y Terapéutica Experimental, Subdirección de Investigaciones Clínicas, Instituto Nacional de Psiquiatría, Ciudad de México, Mexico

**Keywords:** active vaccination, cocaine, antibodies, COC-TT vaccine, cocaine self-administration, cocaine place preference

## Abstract

**Introduction:**

Active vaccination is an effective therapeutic strategy, capable of decreasing the reinforcing and psychomotor effects of cocaine. Clinical studies have shown that cocaine vaccines show an irregular generation of antibody titers, which are rapidly reduced in the absence of reimmunization. The COC-TT vaccine has demonstrated, in rodents, the production of high levels of anti-cocaine antibodies, capable of reducing the cocaine-reinforcing effects, but the adequate dose to obtain the highest antibody titers has not yet been determined, as well as the kinetics of the decay of titers and the capacity to decrease the locomotor activity induced by different doses of cocaine during the phase of decay of titers, induction and expression of locomotor sensitization. The objective of this study was to determine the optimal dose of the COC-TT vaccine, the decay kinetics of anti-cocaine titers, and the efficacy of the antibodies to decrease the locomotor activity induced by different doses of cocaine.

**Methods:**

Male Wistar rats were immunized with the COC-TT. A solid-phase antibody-capture ELISA was used to monitor antibody titer responses after each booster dose in vaccinated animals. The study used cocaine-induced locomotor activity testing to evaluate the cocaine-psychomotor effects.

**Results:**

The COC-TT vaccine could generate high levels of anti-cocaine antibodies. These showed a gradual, dose-dependent decay kinetics of the COC-TT vaccine and a rapid recovery in antibody levels after re-immunization. Furthermore, the antibodies attenuated cocaine-induced locomotor activity during the induction and expression of locomotor sensitization.

**Discussion:**

These findings suggest that the COC-TT vaccine generates a robust immunogenic response capable of reducing the reinforcing effects of cocaine, which supports its possible future use in clinical trials in patients with CUD.

## 1 Introduction

One of the most promising therapeutic strategies in the field of cocaine use disorder (CUD) is active vaccination (1, 2). To date, various vaccines against drugs such as cocaine (3), nicotine (4), heroin (5), and other opioid drugs (6) have been developed and evaluated for their efficacy, with relative success in animals (7) and even in primates (8) and humans (9).

However, the main limitation of the use of this therapeutic strategy in clinical trials is the irregularity of antibody production (10–13). Clinical studies indicate that this depends on everyone, and mention that a large percentage of these vaccinated patients show modest specific antibody titers, with a very rapid decay curve (9–15).

In the case of cocaine, a drug with a high addictive potential (16, 17), several vaccines have been developed with relative success (18–23). These studies demonstrated that the anti-cocaine antibodies generated by these vaccines can prevent cocaine from crossing the blood-brain barrier (BB; 24-27) and decreased cocaine-induced self-administration (23–26, 28–31), place-preference (32), and locomotor activity (20, 21, 26, 33–40), in rodents.

Since human studies suggest that the production of specific anti-cocaine antibodies is irregular and modest, and antibody titers decay very quickly (9–15), a vaccine should be sought that generates not only a large number of specific antibodies, but must also demonstrate that antibody titers do not depend on constant immunization, i.e., it must show a slow decay curve, such that the antibodies can protect the patient for long periods; furthermore, it must demonstrate that re-immunization can recover antibody levels to levels similar to those shown during the vaccination period, and finally, antibody titers must be sufficient to decrease the behavioral effect of different doses of cocaine.

In this regard, in our laboratory, we previously reported the efficacy of a cocaine-tetanus toxoid conjugate vaccine (COC-TT; 3). This vaccine generated a robust immunogenic response, characterized by high levels of anti-cocaine antibodies (1: 600,000). These specific antibodies were able to reduce the cocaine-reinforcing effects, characterized by a decrease in cocaine self-administration, cocaine place preference, and cocaine-induced Fos protein expression, in rats (3).

It is important to mention that the COC-TT vaccine is the optimization of the tetanus toxoid-conjugated morphine vaccine (M_6_-TT; 41–44).

In this sense, the M_6_-TT vaccine could generate high titers of antibodies capable of capturing morphine, heroin, and their metabolites (5, 41); furthermore, the studies by Anton et al., 2006, showed that antibody levels generated by the M_6_-TT vaccine slowly decline and recover to values like those of the last immunization after a re-boost (5). Thus, it is likely that the COC-TT vaccine will show an immunogenic capacity like the M_6_-TT vaccine, which would make it a good candidate for clinical use.

The aim of this study was 1) to determine the decay curve of anti-cocaine antibody titers generated by immunization with the COC-TT vaccine. 2) to determine whether antibody titers displayed during the decay phase of antibody titers decreased cocaine-induced locomotor activity during the decay phase. 3) to determine whether antibodies generated by the COC-TT vaccine attenuate locomotor activity induced by different doses of cocaine during the induction and expression phase of locomotor sensitization and 4) to determine whether cocaine-specific antibodies generated by the COC-TT vaccine suppress the motor effects induced by binge cocaine administration.

## 2 Methods

### 2.1 Subjects

Male *Wistar* rats (250-280 g) were used in this study. Four per cage were housed in standard plastic rodent cages (57 cm X 35 cm X 20 cm) in a colony room at 21 ± 2°C. and 40-50% humidity, under a 12-h light/dark cycle (lights on at 7:00 AM). Food was provided to the animals ad libitum. Experiments were performed during the light phase of the light/dark cycle (9:00 a.m. to 5:00 p.m.). The study was approved by the Animal Care and Bioethics Committee of the National Institute of Psychiatry in Mexico City, in accordance with the Principles of Laboratory Animal Care, as outlined by the National Institutes of Health in the USA.

### 2.2 Drugs

The government of Mexico, under strict regulatory controls, donated cocaine hydrochloride to the National Institute of Psychiatry. The acquisition of the drugs used in this study followed the official standard (COFEPRIS- LC-0004-2003). Before administration, cocaine hydrochloride was prepared. Cocaine was dissolved in sterile saline solutions (0.9% NaCl, Sigma Aldrich) and filtered through a 25-µm syringe filter (Fisher Scientific, Pittsburgh, PA).

### 2.3 Synthesis of the COC-TT vaccine

The synthesis procedure of the COC-TT vaccine was performed according to a method described elsewhere (**Supplementary Material**; 3).

### 2.4 Immunization schedule

Prior to immunization, the TT and COC-TT vaccine were adsorbed onto an aluminum hydroxide gel adjuvant (Pierce, Rockford, IL, USA). 40 rats were immunized with the COC-TT vaccine and 40 rats were immunized with the TT vaccine. Each of the vaccines were administered to each animal subcutaneously (s.c.) at four sites on the shoulders bilaterally (two inoculations/side), for a total vaccine/COC-TT adjuvant dose of 100 µg/1 mg of aluminum hydroxide (≈100 μg/inoculation/animal/booster). To complete the vaccination schedule, animals received 6 booster injections of COC-TT or TT vaccine, using the same unit dose and adjuvant, for 14 to 16 weeks (once every 14 days). Sera were obtained through bleeding, fourteen days after each booster. Subsequently, the serum was frozen at -20 °C until use.

### 2.5 Determination of serum antibody titers via ELISA

A standard protocol was used to perform the determination of serum antibody titers via ELISA (**Supplementary Material**).

### 2.6 Behavioral procedure

#### 2.6.1 Behavioral sensitization procedure

A standard protocol was used to perform the sensitization procedure (44). Briefly, for each animal, locomotor activity was assessed in transparent Plexiglass activity chambers (50x50x30 cm) linked to a PC. Each activity chamber was surrounded by a 16x16 photocell beam array located 3 cm from the floor surface to scan locomotor activity (OMNIALVA, Instruments, Mexico). Photobeam interruptions were automatically quantified with OABiomed software (1.1) and analyses afterward. Locomotor activity was defined as the interruption of consecutive photobeams (OMNIALVA, Mexico).

#### 2.6.2 Procedure

The animals were habituated to the activity chambers in three 30-minute sessions and were randomly assigned to different pharmacological treatment groups. Locomotor activity was recorded for 30 minutes. The rats were returned to their home cages after each experimental session had been completed.

### 2.7 Experimental procedures

The study used 240 male *Wistar* rats, which were assigned to four experimental groups. For Experiment 1, we used 120 animals; 40 animals were used for experiment 2; 40 rats for experiment 3; and 40 animals for experiment 4. Each experimental group received a different pharmacological treatment.

#### 2.7.1 Experiment 1

This experiment focused on determining the dose of the COC-TT vaccine at which the highest anti-cocaine-specific antibody titers were obtained. Additionally, the kinetics of decay and recovery of anti-cocaine antibody titers were characterized in this experiment. In addition, the effectiveness of specific antibody titers in decreasing cocaine-induced locomotor activity during the expression phase of locomotor sensitization, post-immunization (titer decay phase), and recovery (memory) was determined.

This experiment was divided into five experimental phases. Phase I, or the cocaine-induction phase, lasted 10 days. Phase II, or the cocaine-extinction phase, lasted 85 days. This phase includes the immunization phase which consists of 6 immunizations every 14 days. Phase III, or the cocaine-expression phase, lasted 10 days. Phase IV, or the post-immunization phase, lasted 270 days; This includes the determination of the kinetics of decay of specific antibody titers. Phase V, or the memory phase, lasted 60 days. This phase includes a single immunization.

The saline groups received IP sterile saline solution (0.9% NaCl) during the five phases. During the immunization phase, rats in the TT groups received immunization with the TT vaccine, and animals in the COC-TT groups received vaccination with the COC-TT vaccine.

Twelve groups of animals were tested—TT + SAL_-10_ (n = 10); TT + SAL_-40_ (n = 10); TT + COC_-10_ (n = 10); TT + COC_-40_ (n = 10); COC-TT + SAL_-10_ (n = 10); COC-TT + SAL_-40_ (n = 10); COC-TT + SAL_-10_ (n = 10); COC-TT + SAL_-40_ (n = 10); COC-TT_-20μg_ + COC_-10_ (n = 10); COC-TT_-20μg_ + COC_-40_ (n = 10); COC-TT_-50μg_ + COC_-10_ (n = 10); COC-TT_-50μg_ + COC_-40_ (n = 10); COC-TT_-100μg_ + COC_-10_ (n = 10); and COC-TT_-100μg_ + COC_-40_ (n = 10).

The TT + SAL and COC-TT + SAL groups received daily saline administration during the induction, extinction, expression, post-immunization, and memory phases. During the extinction and memory phase, rats in the TT group received immunization with the TT vaccine, and animals in the COC-TT group received vaccination with the COC-TT vaccine (**Table 1**).

**Table 1:** Description of the treatments (doses) that each experimental group received in experiment 1.

GROUP	TREATMENT				
	Induction	Immunization	Expression	Post immunization	Memory
TT + SAL_-10_	Saline	Saline + TT	Saline	Saline	Saline + TT
TT + SAL_-40_	Saline	Saline + TT	Saline	Saline	Saline + TT
TT + COC_-10_	Cocaine_-10mg_	Saline + TT	Cocaine_-10mg_	Cocaine_-10mg_	Cocaine_-10mg_ + TT
TT + COC_-40_	Cocaine_-40mg_	Saline + TT	Cocaine_-40mg_	Cocaine_-40mg_	Cocaine_-40mg_ + TT
COC-TT + SAL_-10_	Saline	Saline + COC-TT	Saline	Saline	Saline + COC-TT
COC-TT + SAL_-40_	Saline	Saline + COC-TT	Saline	Saline	Saline + COC-TT
COC-TT_-20μg_ + COC_-10_	Cocaine_-10mg_	Saline + COC-TT_-20μg_	Cocaine_-10mg_	Cocaine_-10mg_	Cocaine_-10mg_ + COC-TT_-20μg_
COC-TT_-20μg_ + COC_-40_	Cocaine_-40mg_	Saline + COC-TT_-20μg_	Cocaine_-40mg_	Cocaine_-40mg_	Cocaine_-40mg_ + COC-TT_-20μg_
COC-TT_-50μg_ + COC_-10_	Cocaine_-10mg_	Saline + COC-TT_-50μg_	Cocaine_-10mg_	Cocaine_-10mg_	Cocaine_-10mg_ + COC-TT_-50μg_
COC-TT_-50μg_ + COC_-40_	Cocaine_-40mg_	Saline + COC-TT_-50μg_	Cocaine_-40mg_	Cocaine_-40mg_	Cocaine_-40mg_ + COC-TT_-50μg_
COC-TT_-100μg_ + COC_-10_	Cocaine_-10mg_	Saline + COC-TT_-100μg_	Cocaine_-10mg_	Cocaine_-10mg_	Cocaine_-10mg_ + COC-TT_-100μg_
COC-TT_-100μg_ + COC_-40_	Cocaine_-40mg_	Saline + COC-TT_-100μg_	Cocaine_-40mg_	Cocaine_-40mg_	Cocaine_-40mg_ + COC-TT_-100μg_

**Figure 1:**
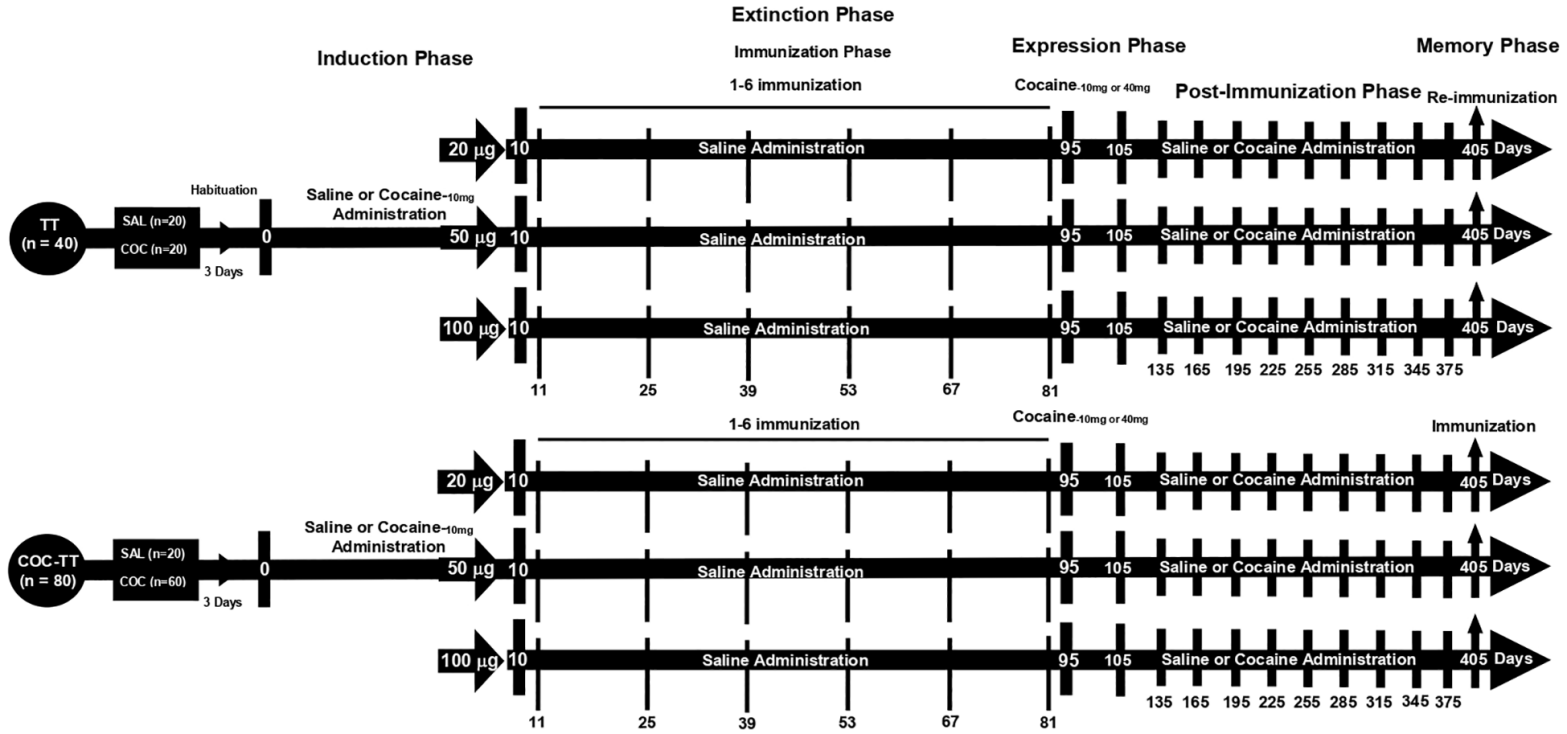
Experiment timeline.

In contrast, the TT + COC and COC-TT + COC groups received cocaine during the induction, expression, post-immunization, and memory phases. In the extinction phase, both groups received daily saline. In the post-immunization and memory phase, animals in the COC-TT_-20μg_ + COC_-10_; COC-TT_-20μg_ + COC_-40_; COC-TT_-50μg_ + COC_-10_; COC-TT_-50μg_ + COC_-40_; COC-TT_-100μg_ + COC_-10_; and COC-TT_-100μg_ + COC_-40_ groups were administered cocaine only every 30 days. During the extinction and memory phase, rats in the TT group received immunization with the TT vaccine, and animals in the COC-TT group received vaccination with the COC-TT vaccine (**Table 1**). 14 days after each immunization serum samples were taken to assess antibody titers. All groups received 6 immunizations, one immunization every 14 days. After each administration, locomotor activity was recorded for 30 minutes for each animal (**Figure 1**).

#### 2.7.2 Experiment 2

**Figure 2:**
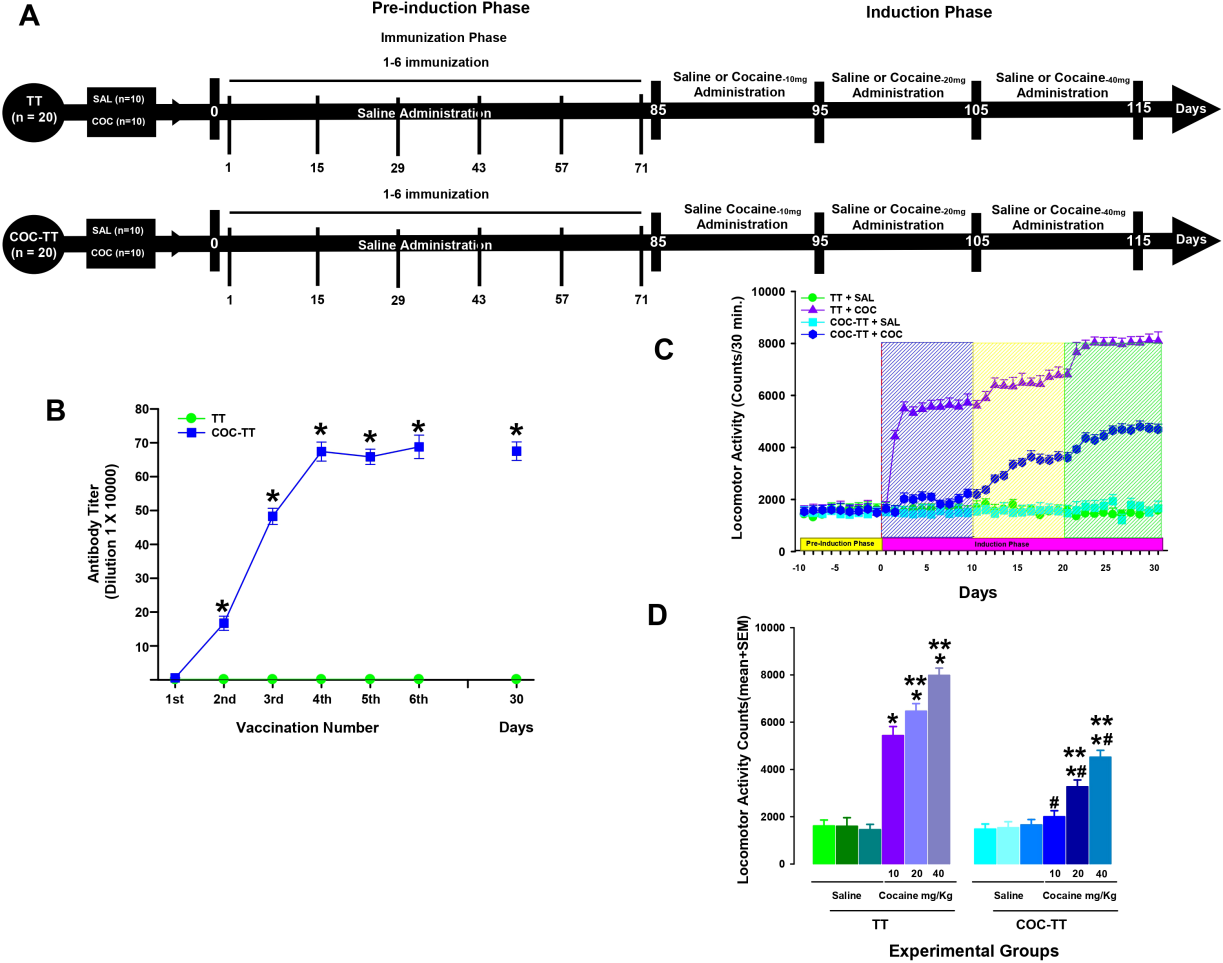
Antibody titer responses (to the sixth boost and 30 days after the last immunization) in rats immunized with the TT or COC-TT vaccine **(B)**. Serum samples were collected 14 days after each immunization. Mean titers (± S.E.M.). *p < 0.01 Significant effects of the antibody titers generated by the COC-TT vaccine to the 6th booster compared to the antibody titers generated by the TT vaccine in Wistar rats. COC-TT vaccine attenuated cocaine-induced locomotor sensitization during the induction phase. Experiment Timeline **(A)**. COC-TT vaccine given for 85 days decreased cocaine-induced locomotor activity **(C)**. Blue shading indicates the 10 days of administration of a 10 mg/kg dose of cocaine; yellow shading indicates administration of 20 mg/kg of cocaine and green shading indicates administration of a 40 mg/kg dose of cocaine during the induction phase. Mean locomotor activity (± S.E.M.) by group (n = 10 animals per group) during the 30 days of the induction phase of locomotor sensitization **(D)**. *p < 0.01 significant effects of the cocaine treatment on locomotor activity, compared to the TT + SAL and the COC-TT + SAL groups. ^#^p < 0.01 significant effects of the COC-TT group compared to the TT + COC group, at different doses of cocaine. **p < 0.01 significant effects between the different doses of cocaine in the TT + COC and COC-TT + COC groups, as determined by three-way ANOVA followed by Tukey’s tests.

This experiment included two pharmacological phases. Phase I, or the pre-induction phase, lasted 85 consecutive days. This phase includes the immunization phase which consists of one immunization every 14 days, totaling 6 immunizations. Phase II, or the cocaine-induction phase, lasted 30 days (**Figure 2A**).

After three days of habituation, the TT + SAL and COC-TT + SAL groups received a saline solution during the above two phases. During the pre-induction phase, rats in the TT groups received immunization with the TT vaccine, and animals in the COC-TT group received vaccination with the COC-TT vaccine.

The TT + COC and COC-TT + COC groups received saline in the pre-induction phase. In the induction phase received different doses of cocaine (10, 20, or 40 mg/Kg). In the pre-induction phase, rats in the TT groups received immunization with the TT vaccine and the animals in the COC-TT group received the vaccination with the COC-TT vaccine. After the administration of each treatment, the animals were immediately placed into the activity chambers, and the locomotor activity of each animal was recorded for 30 minutes (**Figure 2A**).

#### 2.7.3 Experiment 3

**Figure 3:**
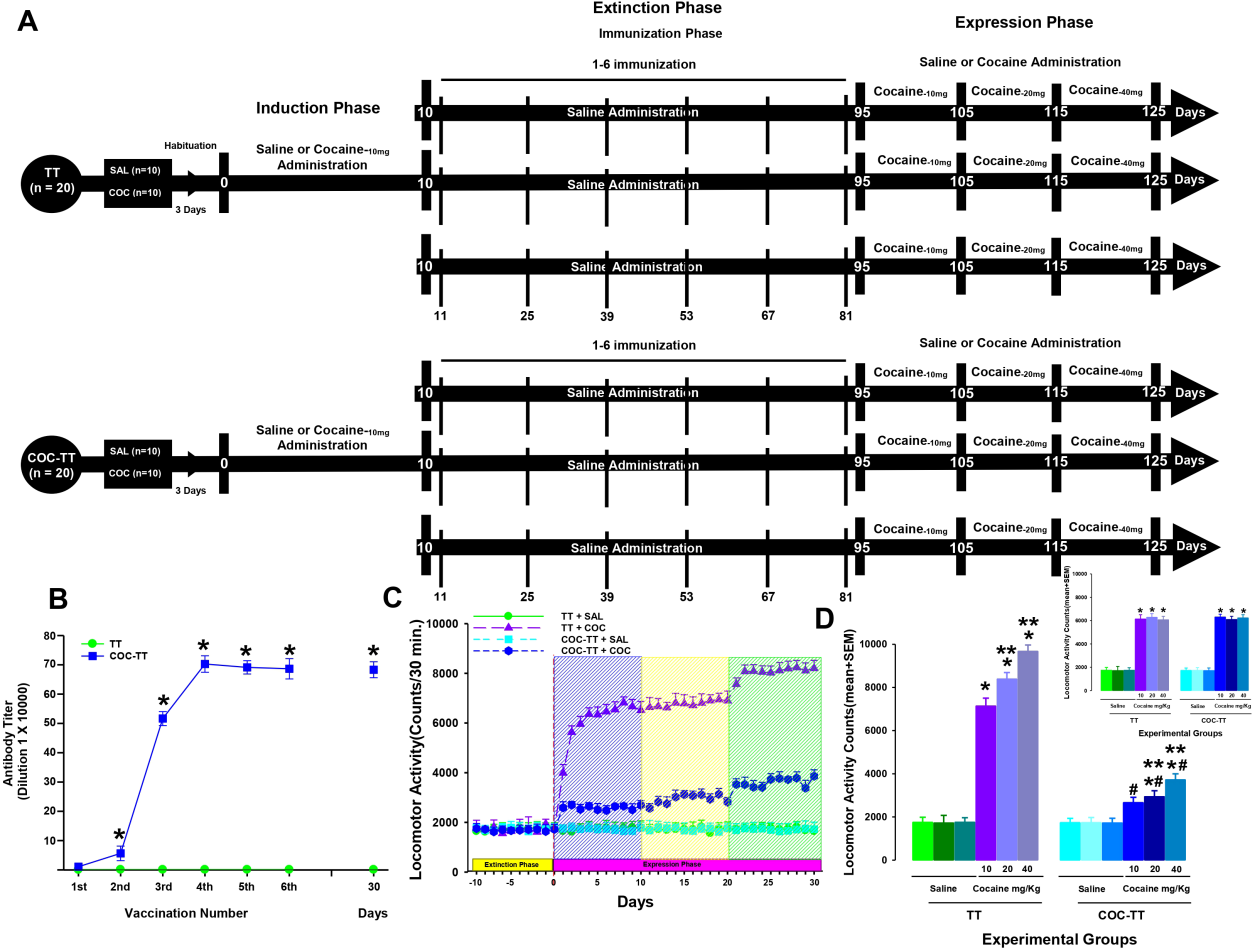
Antibody titer responses (to the sixth boost and 30 days after the last immunization) in rats immunized with the TT or COC-TT vaccine **(B)**. Serum samples were collected 14 days after each immunization. Mean titers (± S.E.M.). *p < 0.01 Significant effects of the antibody titers generated by the COC-TT vaccine to the 6th booster compared to the antibody titers generated by the TT vaccine in Wistar rats. COC-TT vaccine attenuated cocaine-induced locomotor sensitization during the induction phase. Experiment Timeline **(A)**. COC-TT vaccine given for 85 days during the extinction phase decreased cocaine-induced locomotor activity **(C)**. Blue shading indicates the 10 days of administration of a 10 mg/kg dose of cocaine; yellow shading indicates administration of 20 mg/kg of cocaine and green shading indicates administration of a 40 mg/kg dose of cocaine during the induction phase. Mean locomotor activity (± S.E.M.) by group (n = 10 animals per group) during the 30 days of expression phase of locomotor sensitization **(D)**. *p < 0.01 significant effects of the cocaine treatment on locomotor activity, compared to the TT + SAL and the COC-TT + SAL groups. ^#^p < 0.01 significant effects of the COC-TT group compared to the TT + COC group, at different doses of cocaine. **p < 0.01 significant effects between the different doses of cocaine in the TT + COC and COC-TT + COC groups, as determined by three-way ANOVA followed by Tukey’s tests. Inset shows the induction phase inset shows the induction phase *p < 0.01 significant effects of the cocaine treatment on locomotor activity, compared to the TT + SAL and the COC-TT + SAL groups.

This experiment was divided into three experimental phases. Phase I, or the cocaine-induction phase, lasted 10 days. Phase II, or the immunization phase, lasted 85 days. Phase III, or the cocaine-expression phase, lasted 30 days (**Figure 3A**).

The saline groups received IP sterile saline solution (0.9% NaCl) during the three phases. During the immunization phase, rats in the TT groups received immunization with the TT vaccine, and animals in the COC-TT group received vaccination with the COC-TT vaccine.

The animals of the cocaine groups received cocaine (10 mg/Kg) during the induction. During the expression phase, the animals received different doses of cocaine (10, 20, or 40 mg/Kg). In the immunization phase, cocaine was withdrawn and the groups received saline. The rats in the TT groups received immunization with the TT vaccine and the animals in the COC-TT group received the vaccination with the COC-TT vaccine. 14 days after each immunization serum samples were taken to assess antibody titers. All groups received 6 immunizations, one immunization every 14 days. After each administration, locomotor activity was recorded for 30 minutes for each animal (**Figure 3A**).

#### 2.7.4 Experiment 4

**Figure 4:**
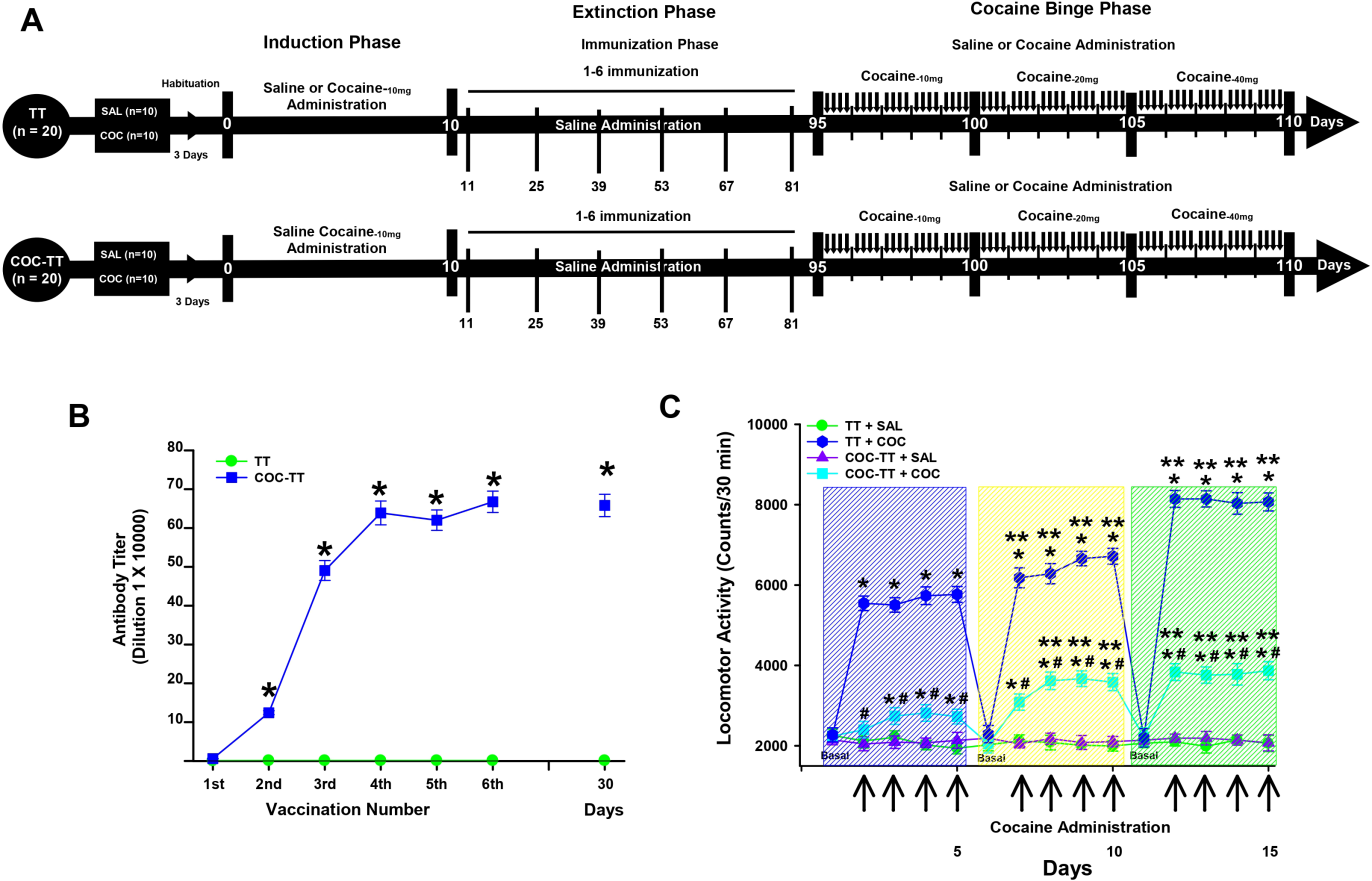
Antibody titer responses (to the sixth boost and 30 days after the last immunization) in rats immunized with the TT or COC-TT vaccine **(B)**. Serum samples were collected 14 days after each immunization. Mean titers (± S.E.M.). *p < 0.01 Significant effects of the antibody titers generated by the COC-TT vaccine to the 6th booster compared to the antibody titers generated by the TT vaccine in Wistar rats. COC-TT vaccine reduced the locomotor activity induced by a binge-like pattern of cocaine administration. Experiment Timeline **(A)**. Mean length (± S.E.M.) of the locomotor effect induced by the treatments **(C)**. *p < 0.01 significant effects of the cocaine treatment on locomotor activity, compared to the TT + SAL and the COC-TT + SAL groups. ^#^p < 0.01 significant effects of the COC-TT group compared to the TT + COC group, at different doses of cocaine. **p < 0.01 significant effects between the doses of cocaine in the TT + COC and COC-TT + COC groups, as determined by three-way repeated-measures ANOVA followed by Tukey’s tests.

Experiment 4 characterized the effect of the COC-TT vaccine on binge-pattern cocaine administration. It included three phases: phase I, cocaine induction, which lasted 10 days; phase II, cocaine extinction or the immunization phase, which lasted 85 days; phase III, or the cocaine binge phase, which lasted 15 days (**Figure 4A**).

During phases I and II, the TT + SAL, the COC-TT + SAL, the TT + COC, and the COC-TT + COC groups received the treatments described in Experiment 3.

In the cocaine binge phase, the TT + SAL and the COC-TT + SAL groups received four daily administrations of sterile saline solution (0.9% NaCl, i.p.), for 5 consecutive days. Each treatment was given every 3 hours for 5 days; the first administration took place at 10:00 A.M.

In the TT + COC and COC-TT + COC groups, the different doses of cocaine (10, 20, 40 mg/Kg) were administered in a binge pattern (4 daily administrations, once every 3 hours) for 5 days; the first administration took place at 10:00 A.M. After the treatments, locomotor activity was recorded for each subject for 30 minutes.

### 2.8 Statistical analysis

Data is expressed as means ± S.E.M. SPSS software version 21.0 was used to perform all statistical analyses. The level of statistical significance was set at p < 0.05.

Experiment 1. Antibody titers were analyzed by one-way repeated measures ANOVA, where the number of vaccinations (1-6 boost) was repeated measures, and the treatments (TT or COC-TT) were the between-subjects factors.

Anti-cocaine antibody levels, in the post-immunization phase, were analyzed by one-way repeated measures ANOVA, where the days post-immunization (270 days) were repeated measures, and the treatments (TT or COC-TT) were the between-subjects factors.

A two-way ANOVA repeated measures were used for analysis with groups (saline and cocaine) and treatment (TT and COC-TT vaccine) between-subject factors and the days post-immunization (270 days) as repeated measures. In both cases, if there was a significant F value in the interaction, a Tukey test was performed for *post hoc* comparisons.

Experiments 2 and 3.

Antibody titers were analyzed by one-way repeated measures ANOVA, where the number of vaccinations (1-6 boost) was repeated measures, and the treatments (TT or COC-TT) were the between-subjects factors.

The results for locomotor activity in each group during the experimental phase were analyzed with a three-way ANOVA used with groups (saline and cocaine), treatment (TT and COC-TT vaccine), and cocaine doses (10, 20, or 40 mg/Kg), as between-subjects factors, followed by a *post hoc* analysis (Tukey’s test).

Experiment 4.

Antibody titers were analyzed by one-way repeated measures ANOVA, where the number of vaccinations (1-6 boost) was repeated measures, and the treatments (TT or COC-TT) were the between-subjects factors.

A three-way ANOVA repeated measures were used for analysis groups (saline and cocaine), treatment (TT and COC-TT vaccine), and cocaine doses (10, 20, or 40 mg/Kg) as between-subject factors, and the cocaine binge days as repeated measures.

## 3 Results

### 3.1 Experiment 1

**Titers**

**Figure 5:**
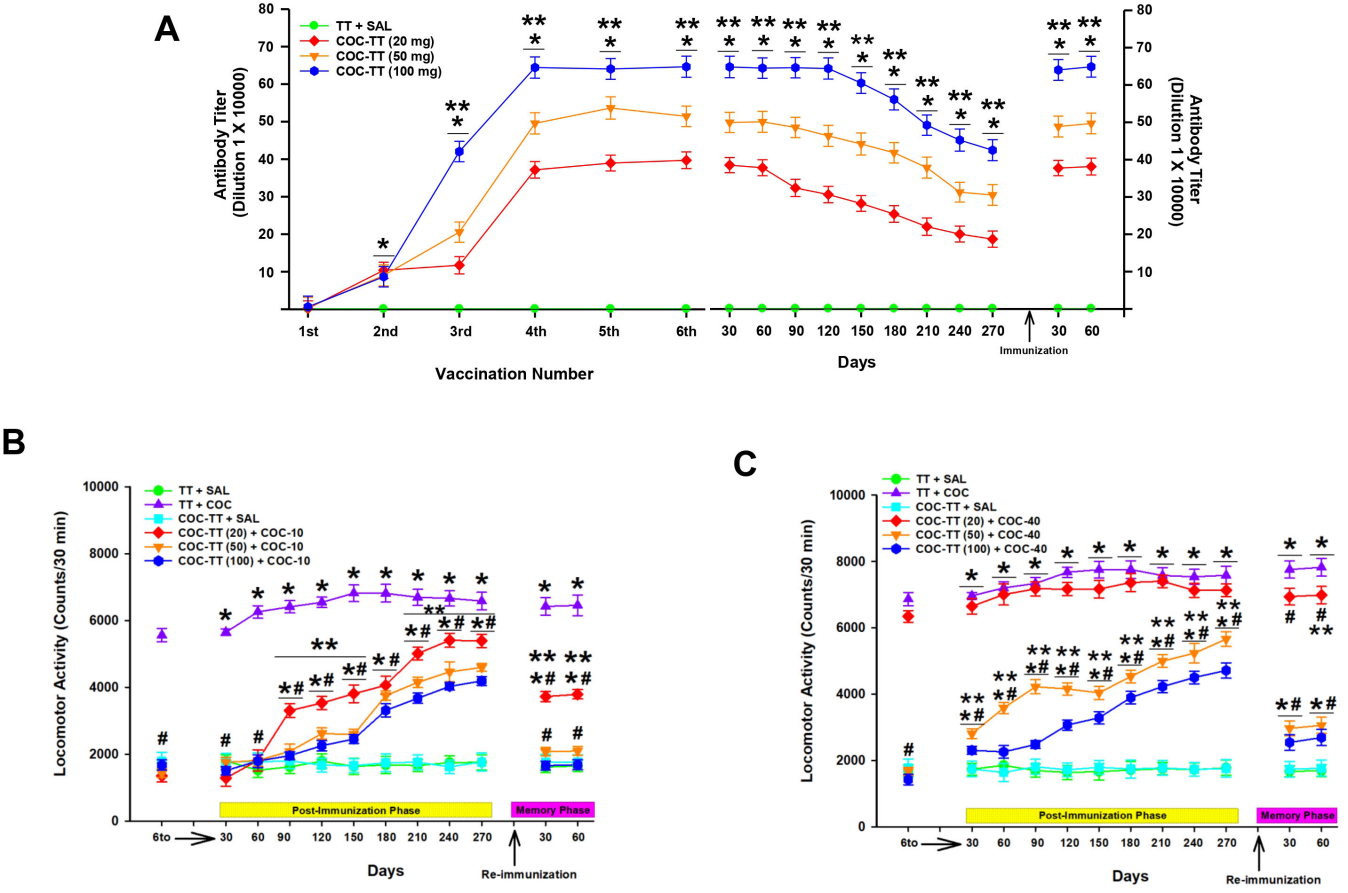
Antibody titer responses (to the sixth boost) in rats immunized with the TT or COC-TT vaccine. Time-course of the kinetics of decay and recovery of titers of the anti-cocaine antibodies **(A)**. Serum samples were collected 14 days after each immunization. Mean titers (± S.E.M.). A slow and progressive decay in antibody levels was observed after the last immunization and later rapidly recovered to maximal levels. *p < 0.01 Significant effects of the antibody titers generated by the COC-TT vaccine to the 6th booster compared to the antibody titers generated by the TT vaccine in Wistar rats. **p < 0.01 significant effects between the different doses of COC-TT + COC. The COC-TT vaccine reduces locomotor activity induced by 10 **(B)** or 40 **(C)** mg/kg of cocaine during the decay and re-immunization phase. Mean locomotor activity (± S.E.M.) by group (n = 10 animals per group). *p < 0.01 significant effects of the cocaine treatment on locomotor activity, compared to the TT + SAL and the COC-TT + SAL groups. ^#^p < 0.01 significant effects of the COC-TT group compared to the TT + COC group, at different doses of cocaine. **p < 0.01 significant effects between the different doses of cocaine in the TT + COC and COC-TT + COC groups, as determined by two-way ANOVA repeated measures followed by Tukey’s tests.

As shown in **Figure 5A**, the TT group immunized with the TT vaccine did not show an increase in anti-cocaine antibody titers. In contrast, in general, the animals vaccinated with the COC-TT vaccine showed a progressive increase (F = (1,39) 89.247, p < 0.001) in anti-cocaine antibody titer measured by ELISA.

Tukey’s test found significant dose-dependent differences in the antibody titers shown by the COC-TT groups compared to the TT (p < 0.001) group, from the second immunization. Furthermore, for the doses of the COC-TT vaccine evaluated (20, 50, or 100 μg), Tukey’s test found that the maximum anti-cocaine antibody titer for the COC-TT groups was reached after the fourth booster (p < 0.001).

Additionally, *post hoc* analysis revealed differences in anti-cocaine antibody titers shown by the COC-TT-_100μg_ + COC group compared to the antibody level shown by the COC-TT_-20μg_ + COC (p < 0.001) and COC-TT-_50μg_ + COC (p < 0.002) groups from the third immunization. Furthermore, the statistical analysis also found differences in anti-cocaine-specific antibody titers between the COC-TT_-50μg_ + COC and COC-TT_-20μg_ + COC (p < 0.002) groups (**Figure 5A**).

Subsequently, the progressive decay in anti-cocaine antibody titers in non-boosted immunized animals was characterized. Antibody titers showed a significant dose- and time-dependent (days) decay, reaching their lowest levels 270 days after the last immunization (F = (1,36) 240.058, p < 0.001). After the significant decay in antibody titers, a rapid recovery to peak levels was observed 30 days after a re-immunization.

Specific antibody titers displayed by the COC-TT_-20μg_ + COC group showed a significant decay from the first 30 days after the last immunization compared to the decay of antibody titers displayed by the COC-TT_-50μg_ + COC (p < 0.002) and COC-TT_-100μg_ + COC (p < 0.001) groups. In contrast, the group immunized with 100 μg of COC-TT vaccine showed a slow and gradual decline in titers, showing significant differences from the groups immunized with a dose of 20 (p < 0.001) or 50 μg (p < 0.001) of COC-TT vaccine.

After re-immunization, the antibody levels displayed by the COC-TT groups showed significant differences between the different doses (p < 0.001) but not between antibody levels displayed at the last immunization and those displayed after re-immunization (p = 0.96).

These latest data confirm the efficacy of the COC-TT vaccine in inducing immune humoral memory to cocaine and indicate that the most effective dose of the COC-TT vaccine is 100 μg.

Cocaine locomotor activity―Titers decay―

After the last immunization, 10 or 40 mg/kg of cocaine was administered every 30 days to groups of animals immunized with different doses of the COC-TT vaccine, to associate antibody titers with the decrease in cocaine-induced locomotor activity.

In relation to the 10 mg/kg cocaine dose, animals in the TT + COC_-10_ group showed a sustained increase (two-way repeated measures ANOVA; groups by treatment by doses interaction F= (1, 54) 491.708 p < 0.0001) in cocaine-induced locomotor activity during the 270 days of evaluation compared to the activity shown by the TT + SAL (p < 0.001) and COC-TT + SAL (p < 0.001) groups.

In contrast, groups immunized with different doses of the COC-TT vaccine (20, 50, or 100 μg) showed a gradual increase in cocaine-induced locomotor activity. Tukey’s test found significant differences in cocaine-induced hyperactivity shown by the TT + COC_-10_ group from 30 days onwards compared to the locomotor activity shown by the COC-TT_-20μg_ + COC_-10_ (p < 0.002), COC-TT_-50μg_ + COC_-10_ (p < 0.001), and COC-TT_-100μg_ + COC_-10_ (p < 0.001) groups (**Figure 5B**).

Furthermore, post hoc analysis revealed differences between COC-TT vaccine doses. Tukey's test found significant differences in cocaine-induced locomotor activity shown by the COC-TT_-20μg_ + COC_-10_ group at 90 (p < 0.003), 120 (p < 0.003), 150 (p < 0.002), 210 (p < 0.003), 240 (p < 0.003), and 270 (p < 0.002) days post-immunization compared to cocaine-induced locomotor activity shown by the COC-TT_-50μg_ + COC_-10_, and COC-TT_-100μg_ + COC_-10_ groups. However, statistical analysis found no differences in cocaine-induced hyperactivity between the COC-TT_-50μg_ + COC_-10_, and COC-TT_-100μg_ + COC_-10_ (p = 0.86) groups over the 270 days of evaluation.

As mentioned above, re-immunization restored antibody levels in the groups immunized with 20, 50, or 100 μg of COC-TT vaccine, however, the post hoc test revealed differences in the cocaine-induced locomotor activity shown by the COC-TT_-20μg_ + COC_-10_ group between the last immunization and the re-immunization (p < 0.001). However, no differences were found in the COC-TT_-50μg_ + COC_-10_, and COC-TT_-100μg_ + COC_-10_ (p = 0.98) groups at both phases (**Figure 5B**).

When 40 mg/kg of cocaine was administered, the TT + COC_-40_ and COC-TT_-20μg_ + COC_-40_ groups showed a rapid increase (two-way repeated measures ANOVA; groups by treatment by doses interaction F= (1, 54) 8.393 p < 0.005) in cocaine-induced locomotor activity compared to that shown by the TT + SAL (p < 0.001) and COC-TT + SAL (p < 0.001) groups. In addition, Tukey’s test found significant differences in cocaine-induced hyperactivity shown by the TT + COC_-40_ and COC-TT_-20μg_ + COC_-40_ groups with respect to that shown by the COC-TT_-50μg_ + COC_-40_ (p < 0.001) and COC-TT_-100μg_ + COC_-40_ (p < 0.001) groups over the 270 days of evaluation. However, no difference was found between the TT + COC_-40_ and COC-TT_-20μg_ + COC_-40_ (p = 0.97) groups (**Figure 5C**).

Additionally, the post hoc test found significant differences in the cocaine-induced locomotor activity shown by the COC-TT_-50μg_ + COC_-40_ group compared to that shown by the COC-TT_-100μg_ + COC_-40_ (p < 0.002) group over the 270 days of recording. However, Tukey’s test revealed significant differences in the cocaine-induced locomotor activity shown by the TT + COC_-40_ and COC-TT_-20μg_ + COC_-40_ groups compared to that shown by the COC-TT_-50μg_ + COC_-40_ (p < 0.001) and COC-TT_-100μg_ + COC_-40_ (p < 0.001) groups over the 270 days of evaluation.

After re-immunization, statistical analysis found no differences in cocaine-induced hyperactivity shown by the COC-TT_-20μg_ + COC_-40_ (p = 0.92) group during the last immunization compared to that shown at re-immunization. However, it did reveal differences in locomotor activity shown by the COC-TT_-50μg_ + COC_-40_ (p < 0.003) and COC-TT_-100μg_ + COC_-40_ (p < 0.003) groups between the last immunization and the re-immunization (**Figure 5C**).

### 3.2 Experiment 2

Titers

TT group immunized with the TT vaccine did not show an increase in anti-cocaine antibody titers. In contrast, the animals immunized with the COC-TT vaccine showed a progressive increase (F = (1, 19) 18.644, p < 0.001) in anti-cocaine antibody titer measured by ELISA (**Figure 2B**).

Tukey’s test found significant differences in the antibody titers shown by the COC-TT groups compared to the TT (p < 0.001) group, from the second immunization. Additionally, Tukey’s test found that the maximum anti-cocaine antibody titer for the COC-TT groups was reached after the fourth booster (p < 0.001).

Cocaine locomotor activity―Induction―

The TT+ SAL and the COC-TT + SAL groups showed no significant increases in locomotor activity (p = 0.99). As shown in **Figure 2C**, the injection of different doses of cocaine (10, 20, or 40 mg/Kg) generates a dose-dependent increase in cocaine-induced locomotor activity during induction (three-way ANOVA; in the groups by treatment by doses interaction, F (2,120) = 16.286, p < 0.0001) phase compared to the TT + SAL (p <0.001) and the COC-TT + SAL (p <0.001) groups. In addition, the *post hoc* test found no differences in locomotor activity between the TT + SAL and COC-TT + SAL groups (p = 0.96).

Tukey’s test revealed significant differences in locomotor activity induced by a dose of 10 mg/kg compared to hyperactivity induced by a dose of 20 (p <0.003) or 40 (p <0.001) mg/kg of cocaine; it also found differences between locomotor activity induced by 20 and 40 (p <0.002) mg/kg of cocaine, during the induction phase (**Figure 2D**).

In contrast, the COC-TT group showed a long-term attenuation in cocaine-induced locomotor activity in the induction phase compared to the TT + COC group. In this sense, the *post-hoc* analysis found differences in the locomotor activity induced by 10, 20, or 40 mg/kg of cocaine shown by the COC-TT group with respect to that shown by the TT + COC group, at the corresponding doses (p < 0.001). **Figure 2D** showed that Tukey’s test did not find differences in the locomotor activity induced by the administration of 10 mg/kg of cocaine to the COC-TT group compared to that shown by the COC-TT + SAL (p = 0.94) and TT + SAL (p = 0.97) groups. In contrast, a dose of 20 or 40 mg/kg of cocaine generated differences in cocaine hyperactivity with respect to the TT + SAL (p < 0.002) and COC-TT + SAL (p < 0.002) groups.

In addition, statistical analysis revealed dose-dependent differences in the COC-TT + COC group when comparing locomotor activity induced by 20 or 40 mg/kg cocaine with hyperactivity induced by 10 mg/kg cocaine; the *post-hoc* test found significant differences (p < 0.002). Furthermore, the Tukey test revealed differences in the COC-TT + COC group between doses of 20 and 40 mg/kg (p < 0.003) cocaine (**Figure 2D**). These findings suggest that the COC-TT vaccine produces a long-term attenuation of the induction of cocaine locomotor sensitization generated by different doses of cocaine.

### 3.3 Experiment 3

Titers

TT group immunized with the TT vaccine did not show an increase in anti-cocaine antibody titers. In contrast, the animals immunized with the COC-TT vaccine showed a progressive increase (F = (1,19) 18.640, p < 0.001) in anti-cocaine antibody titer measured by ELISA (**Figure 3B**).

Tukey’s test found significant differences in the antibody titers shown by the COC-TT groups compared to the TT (p < 0.001) group, from the second immunization. Additionally, Tukey’s test found that the maximum anti-cocaine antibody titer for the COC-TT groups was reached after the fourth booster (p < 0.001).

Cocaine locomotor activity―Expression―

As shown in the above experiment, cocaine significantly increased locomotor activity during the induction (Three-way ANOVA; groups by treatment by doses interaction; F(2, 120) = 0.011 p = 0.98) phase.

In this experimental phase, Tukey’s test revealed differences in locomotor activity when the TT + COC and COC-TT + COC groups were compared to the TT + SAL (p < 0.0001) and COC-TT + SAL (p < 0.0001) groups.

As shown in **Figure 3C**, a dose of 10 mg/Kg, 20 mg/Kg, or 40 mg/Kg of cocaine significantly increased locomotor activity during the expression (Three-way ANOVA; groups by treatment by doses interaction; F(2, 120) = 26.834 p < 0.001) phase in the rats immunized with the TT vaccine compared to the TT + SAL (P < 0.001) and COC-TT + SAL (P < 0.001) groups.

Statistical analysis revealed significant differences in the cocaine-induced locomotor activity shown by the TT + COC group dosed with 10 mg/Kg of cocaine compared to when this group was administered 20 or 40 mg/Kg of cocaine (p < 0.002). Additionally, Tukey’s test revealed differences in hyperactivity induced by 20 mg/kg cocaine compared to that generated by 40 mg/kg (p < 0.002) cocaine (**Figure 3D**). In contrast, the *post hoc* test found no differences in locomotor activity between the TT + SAL and COC-TT + SAL groups (p = 0.98).

Regarding the group immunized with the COC-TT vaccine; daily administration of different doses of cocaine (10, 20, or 40 mg/kg) to rats previously vaccinated with the COC-TT vaccine did not increase locomotor activity significantly (**Figure 3D**).

Tukey’s test found significant differences in the locomotor activity induced by 10, 20, and 40 mg/Kg of cocaine in the COC-TT + COC group compared to the locomotor activity shown by the TT + COC group dosed with 10 mg/Kg (p < 0.001), 20 mg/Kg (p < 0.001), or 40 mg/Kg (p < 0.001) of cocaine (**Figure 3D**).

Furthermore, *post-hoc* analysis found differences in locomotor activity induced by the different doses of cocaine in the COC-TT + COC group compared to the locomotor activity shown by the TT + SAL (p < 0.002) and COC-TT + SAL (p < 0.002) groups.

Finally, Tukey’s test revealed differences in the cocaine-induced locomotor activity shown by the COC-TT + COC group when dosed with 20 mg/Kg of cocaine compared to the locomotor activity induced by 40 mg/Kg (p < 0.002) of cocaine. But no differences were found between 10 mg/kg and 20 mg/kg (p = 0.95) of cocaine (**Figure 3D**).

This finding suggests that the vaccination with the COC-TT vaccine during the extinction phase results in long-term attenuation of the expression of cocaine-induced locomotor sensitization.

### 3.4 Experiment 4

**Titers**

TT group immunized with the TT vaccine did not show an increase in anti-cocaine antibody titers. In contrast, the animals immunized with the COC-TT vaccine showed a progressive increase (F = 1,19) 18.601, p < 0.001) in anti-cocaine antibody titer measured by ELISA (**Figure 4B**).

Tukey test found significant differences in the antibody titers shown by the COC-TT groups compared to the TT (p < 0.001) group, from the second immunization. Additionally, the Tukey test found that the maximum anti-cocaine antibody titer for the COC-TT groups was reached after the fourth booster (p < 0.001).

Cocaine binge

Experiment 4 characterized the effect of the COC-TT vaccine on binge-pattern cocaine administration. Vaccination with the COC-TT vaccine decreases the locomotor activity caused by a binge-like pattern of cocaine administration (three-way repeated measures ANOVA; groups by treatment by doses interaction F= (1, 108) 14.161 p < 0.0001).

During the administration of 10 mg/kg of cocaine, the statistical analysis did not find differences in the locomotor activity shown by the COC-TT + COC group compared to the activity shown by the TT + SAL (p = 0.98) and COC-TT + SAL (p = 0.75) group, during the first and second cocaine administration. However, from the third administration, the Tukey’s test revealed significant differences in locomotor activity between the COC-TT + COC group and the TT + SAL (p < 0.002) and the COC-TT + SAL (p < 0.002) groups (**Figure 4C**).

Additionally, at a dose of 20 and 40 mg/kg of cocaine, the *post hoc* test found differences in the COC-TT + COC group from the first administration with respect to the TT + SAL (p < 0.001) and COC-TT + SAL (p < 0.001) groups.

Additionally, the *post hoc* test found differences between the COC-TT + COC and TT + COC groups from the first administration of cocaine (p < 0.001) at a dose of 10, 20, or 40 mg/Kg of cocaine (**Figure 4C**).

These results suggested that the cocaine vaccine was able to significantly decrease locomotor activity induced by a binge-pattern cocaine administration.

## 4 Discussion

### Immune response

In this study, we found a) dose-dependent differences in anti-cocaine antibody levels at the sixth immunization; b) different dose-dependent decay kinetics of the COC-TT vaccine; c) we found that the speed of recovery of antibody titers at each dose of COC-TT vaccine evaluated was similar; and d) a gradual dose-dependent increase in locomotor activity induced by different doses of cocaine, during the decay kinetics evaluation stage, dependent on COC-TT vaccine dose.

As shown in **Figure 5A**, at all three COC-TT vaccine doses tested, anti-cocaine antibody titers showed a gradual increase, reaching anti-cocaine antibody levels in the range of ~1:350,000 to 1:650,000 by the sixth immunization depending on the COC-TT vaccine dose tested. These results are in line with previous reports indicating that rodents immunized with 20 or 50 μg― as the lower dose― and 200 μg― as the upper dose― of PDD-GNE or dAd5-GNE vaccines showed a gradual, vaccine dose-dependent increase in antibody levels, reaching peak antibody levels at a vaccine dose of 200 μg (21, 26, 27, 31, 32, 45). However, differences in anti-cocaine antibody titers were found between the GNE-FLiC and COC-TT vaccines. In the case of the GNE-FLiC vaccine, the maximum antibody levels were reached at a dose of 50 μg (47, 48), whereas the maximum antibody titers produced by the COC-TT vaccine were reached at a dose of 100 μg.

On the other hand, differences in anti-cocaine antibody titers shown at the last immunization between the COC-TT vaccine and the pioneer anti-cocaine vaccines, at equivalent doses, which were synthesized with conventional carrier proteins such as BSA or KLH, were found. Vaccines such as GNC-KLH (34), COC-BSA (23), and SNC-KLH38 (46), showed anti-cocaine antibody titers of ≈ 1: 15,000 to 1: 80,000. Even a vaccine such as SNC-rCTB, which was evaluated in humans, showed antibody levels of ≈1: 2,400 (13).

Thus, the difference in the vaccine dose at which the maximum antibody level is achieved is likely due to the carrier protein from which the vaccine was synthesized. In fact, the development of a new generation of cocaine vaccines called self-adjuvating cocaine vaccines has recently been reported. These vaccines were synthesized with new generation carrier proteins—dendrimeric peptides, adenoviruses, or nanoproteins (3, 21, 26, 27, 46–48), which were able to generate high antibody titers (8, 19, 20, 32, 47).

The dAd5GNE and dAd5GNC vaccines, for example, were synthesized with an adenovirus as carrier proteins, which allowed expands the number of hapten binding sites, generated a range of anti-cocaine antibody titers from 1: 300,000 to 1: 900,000 (21, 26, 46). Instead, the conjugates GNE-FliC, CocKFE8, GNE-PDD, and GNE-calix[n]arene (UFMG-V4N2) were constructed from nano scaffolds or nanoparticle platforms (flagellin, KFE8 peptide, PDD dendrimers, and calix[n]arene). Immunization with these vaccines generated high levels of anti-cocaine antibodies, in the range of ≈ 1:500,000 to 1:700,000 (8, 19, 20, 32, 47). However, an important limitation of the use of these nanoparticle platforms is the long regulatory path that they must comply with to be used in any clinical trial. This limitation is not present in the COC-TT vaccine and all vaccines synthesized with tetanus toxoid (33, 48, 49), a carrier protein approved for human use.

Regarding the decay kinetics, to our knowledge there are no reports on this matter; this would be the first scientific report showing the decay kinetics of anti-cocaine antibody titers.

This suggests that 100 μg of COC-TT vaccine produces the maximum level of anti-cocaine antibodies and may be considered the optimal dose in immunization protocols in future preclinical and clinical studies. This observation is consistent with experimental reports indicating that the anti-cocaine vaccine dose generally used in immunization protocols is 100 μg (8, 14, 20, 25, 31, 38, 45, 50).

What we found in this study was a gradual decrease, dependent on the dose of the COC-TT vaccine, in the levels of anti-cocaine antibodies. A dose of 20 μg of the COC-TT vaccine shows a faster decay kinetics than that found at a dose of 100 μg of the COC-TT vaccine, where the decay kinetics are very slow. The differences found between the doses of the COC-TT vaccine could be due to the amount of haptens that were coupled to the carrier protein, however, other types of studies are required to determine the haptenization index and confirm this hypothesis. The important thing is that this result suggests that the COC-TT vaccine generates long-term protection not dependent on constant re-immunizations.

Another important observation, and one for which we found no previously reported evidence, is related to the speed with which re-immunization induces the production of anti-cocaine antibodies. This was not dose-dependent; the three doses of the COC-TT vaccine reached maximum antibody levels like those observed at the sixth immunization, at the same speed (14 days after immunization), suggesting that the COC-TT vaccine is effective in inducing a humoral immune memory response to cocaine.

Regarding the effectiveness of anti-cocaine antibody titers during the decay phase, at a dose of 10 mg/kg of cocaine, the antibody levels produced by the three doses (20, 50, or 100 μg) of COC-TT vaccine evaluated were sufficient to significantly decrease long-term cocaine-induced locomotor activity. This suggests that anti-cocaine antibody titers in a range of ≈ 1:200,000 to 1:450,000 are sufficient to decrease hyperlocomotion induced by 10 mg/kg of cocaine. However, this range of antibody titers was not effective in decreasing locomotor activity induced by 20 mg/kg of cocaine. As shown in **Figure 5C**, the level of anti-cocaine antibodies elicited by a 20 μg dose of COC-TT vaccine (≈ 1:200,000 to 1:300,000) was not effective in attenuating locomotor activity induced by 20 mg/kg of cocaine. In contrast, anti-cocaine titers ranging from ≈ 1:350,000 to 1:600,000 were effective in reducing hyperlocomotion induced by 20 mg/kg of cocaine.

We have not found similar reports in the literature, so this is the first time that the long-term effectiveness of a cocaine vaccine in attenuating locomotor activity induced by different doses of cocaine has been reported. And suggests that an anti-cocaine antibody window of ≈ 1:350,000 to 1:600,000 is sufficient and effective to decrease locomotor activity induced by doses of up to 20 mg/kg of cocaine. And supports the use of a dose of 100 μg of COC-TT vaccine, as the dose that induces the best immunological and immunoprotective effects.

### Cocaine locomotor sensitization

As shown in **Figures 2**, **3**, immunization with 100 μg of COC-TT vaccine produced anti-cocaine antibody levels in the range of ≈ 1:650,000 to 1:700,000. This result is in line with anti-cocaine antibody levels generated with tetanus toxoid-based vaccines. Kimishima et al. showed that their GNE-TT and GND-TT vaccines generated antibody levels in the range of ≈ 1:400,000 to 1:700,000 (33, 48).

Pioneering studies reported that anti-cocaine antibody levels induced by the GNC-KLH vaccine (≈ 1:25,000) were effective in decreasing locomotor activity induced by 15 mg/kg cocaine (34, 35). Similarly, vaccination of rodents with the SNC-KLH, GNF-KLH, GNCF-KLH, GNE−KLH, GNT−KLH, and GNE-FliC vaccines, which produced antibody levels ranging from 1:13,000 to 1:30,000, decreased locomotor activity induced by 5 or 10 mg/kg cocaine (36, 37, 40, 51, 52). On the other hand, vaccines producing antibody titers between 1:120,000 to 1:720,000 such as dAd5GNE, dAd5GNC, or SNC-rCTB vaccines were able to decrease hyperlocomotor activity induced by 15 mg/kg of cocaine (21, 26, 38). In this sense, the decrease in locomotor activity induced by 10 mg/kg of cocaine, described in this study, is consistent with the results previously described by these studies.

Interestingly, Kimishima et al. reported that immunization of mice with the GND-TT and GNE-TT vaccines, which produced anti-cocaine antibody titers in the range of 1:200,000 to 1:350,000, showed a decrease in locomotor activity induced by 10 and 20 mg/kg of cocaine (33, 48). These results are in line with the results reported in this study, where rats vaccinated with the COC-TT vaccine showed a significant decrease in the locomotor effect induced by 10 or 20 mg/kg of cocaine. It suggests that a very important structural element of the GND-TT, GNE-TT, and COC-TT vaccines to induce high levels of anti-cocaine antibodies capable of decreasing the locomotor effects induced by different doses of cocaine is the carrier protein (tetanus toxoid). This hypothesis is supported by the observation found in this study where the anti-cocaine antibody titers produced by the COC-TT vaccine are effective in decreasing the locomotor activity induced by cocaine doses of up to 40 mg/kg. This result is also novel since we have not found any reports in the literature indicating that the anti-cocaine antibody titers produced by any cocaine vaccine can decrease the hyper locomotor activity induced by doses higher than 20 mg/kg of cocaine.

Another important fact, which is also not referenced in the literature, with which to compare the efficacy of the COC-TT vaccine, is the efficacy shown by the anti-cocaine antibodies produced by the COC-TT vaccine to decrease the induction and expression of locomotor sensitization induced by different doses of cocaine (10, 20 or 40 mg/kg cocaine).

In this sense, the fact that the COC-TT vaccine can attenuate both phases (induction and expression) of locomotor sensitization and given that behavioral sensitization has been shown to be an important factor in the development of drug addiction because of its important role in the relapse in drug use (53, 54), then suggests that the COC-TT vaccine could decrease the percentages of relapses to cocaine use and supports its possible use in humans.

### Cocaine binge

Another very important piece of evidence, which we also found no evidence for in the literature, is the observation that anti-cocaine antibodies produced by immunization with the COC-TT vaccine decreased locomotor activity induced by a binge pattern of cocaine administration.

Cocaine is frequently consumed in intermittent cycles of repeated dosing, or “binges,” which produce changes in mood and cocaine craving and cardiovascular and subjective effects (55–57). Additionally, it has been reported that escalation of a pattern of cocaine binge administration in rats and humans produces obvious signs of psychosis, and toxicity, including ataxia, convulsions, lethargy, and eventual death (58–60). Thus, an additional therapeutic advantage provided by the COC-TT vaccine would be its ability to reduce cocaine-induced psychosis and toxicity in binge administration and could even reduce the deaths caused by the toxic effects of cocaine consumption during binge administration.

However, to validate its use in humans, further preclinical, toxicity, and biological safety studies are still required, but the evidence presented in this study supports its possible use in clinical protocols.

## Data Availability

The raw data supporting the conclusions of this article will be made available by the authors, without undue reservation.

## References

[1] Kosten TR. Vaccines as immunotherapies for substance use disorders. Am J Psychiatry. (2024) 181:362–71. doi: 10.1176/appi.ajp.20230828 38706331

[2] Lu T Li X Zheng W Kuang C Wu B Liu X. Vaccines to treat substance use disorders: current status and future directions. Pharmaceutics. (2024) 16:84. doi: 10.3390/pharmaceutics16010084 38258095 PMC10820210

[3] Barbosa-Méndez S Matus-Ortega M Hernandez-Miramontes R Salazar-Juarez A. COT-TT vaccine attenuates cocaine-seeking and cocaine-conditioned place preference in rats. Hum Vaccin Immunother. (2024) 20:2299068. doi: 10.1080/21645515.2023.2299068 38228468 PMC10793666

[4] Keyler DE Roiko SA Earley CA Murtaugh MP Pentel PR. Enhanced immunogenicity of a bivalent nicotine vaccine. Int Immunopharmacol. (2008) 8:1589–94. doi: 10.1016/j.intimp.2008.07.001 PMC2577591 18656557

[5] Anton B Leff P. A novel bivalent morphine/heroin vaccine that prevents relapse to heroin addiction in rodents. Vaccine. (2006) 24:3232–40. doi: 10.1016/j.vaccine.2006.01.047 16494974

[6] Haile CN Baker MD Sanchez SA Lopez Arteaga CA Duddupudi AL Cuny GD. An immunconjugate vaccine alters distribution and reduces the antinociceptive, behavioral and physiological effects of fentanyl in male and female rats. Pharmaceutics. (2022) 14:2290. doi: 10.3390/pharmaceutics14112290 36365109 PMC9694531

[7] Bagasra O Forman LJ Howeedy A Whittle P. A potential vaccine for cocaine abuse prophylaxis. Immunopharmacology. (1992) 23:173–9. doi: 10.1016/0162-3109(92)90023-6 1500284

[8] Sabato B Augusto PSA Lima Gonçalves Pereira R Coutinho Batista Esteves F Caligiorne SM Rodrigues Dias Assis B. Safety and immunogenicity of the anti-cocaine vaccine UFMG-VAC-V4N2 in a non-human primate model. Vaccine. (2023) 41:2127–36. doi: 10.1016/j.vaccine.2023.02.031 36822966

[9] Kosten TR Domingo CB Shorter D Orson F Green C Somoza E. Vaccine for cocaine dependence: a randomized double-blind placebo-controlled efficacy trial. Drug Alcohol Depend. (2014) 140:42–7. doi: 10.1016/j.drugalcdep.2014.04.003 PMC4073297 24793366

[10] Orson FM Rossen RD Shen X Lopez AY Wu Y Kosten TR. Spontaneous development of IgM anti-cocaine antibodies in habitual cocaine users: effect on IgG antibody responses to a cocaine cholera toxin B conjugate vaccine. Am J Addict. (2013) 22:169–74. doi: 10.1111/j.1521-0391.2013.00314.x PMC4143984 23414504

[11] Haney M Gunderson EW Jiang H Collins ED Foltin RW. Cocaine-specific antibodies blunt the subjective effects of smoked cocaine in humans. Biol Psychiatry. (2010) 67:59–65. doi: 10.1016/j.biopsych.2009.08.031 19846066 PMC3319755

[12] Martell BA Orson FM Poling J Mitchell E Rossen RD Gardner T. Cocaine vaccine for the treatment of cocaine dependence in methadone-maintained patients: a randomized, double-blind, placebo-controlled efficacy trial. Arch Gen Psychiatry. (2009) 66:1116–23. doi: 10.1001/archgenpsychiatry.2009.128 PMC2878137 19805702

[13] Kosten TR Rosen M Bond J Settles M Roberts JS Shields J. Human therapeutic cocaine vaccine: safety and immunogenicity. Vaccine. (2002) 20:1196–204. doi: 10.1016/s0264-410x(01)00425-x 11803082

[14] Kosten TR Domingo CB Hamon SC Nielsen DA. DBH gene as predictor of response in a cocaine vaccine clinical trial. Neurosci Lett. (2013) 541:29–33. doi: 10.1016/j.neulet.2013.02.037 23458673 PMC3631430

[15] Nielsen DA Hamon SC Kosten TR. The κ-opioid receptor gene as a predictor of response in a cocaine vaccine clinical trial. Psychiatr Genet. (2013) 23:225–32. doi: 10.1097/YPG.0000000000000008 PMC3885873 23995774

[16] Schwartz EKC Wolkowicz NR De Aquino JP MacLean RR Sofuoglu M. Cocaine use disorder (CUD): current clinical perspectives. Subst Abuse Rehabil. (2022) 13:25–46. doi: 10.2147/SAR.S337338 36093428 PMC9451050

[17] Li MJ Shoptaw SJ. Clinical management of psychostimulant withdrawal: review of the evidence. Addiction. (2023) 118:750–62. doi: 10.1111/add.v118.4 PMC10069411 36401591

[18] Madge HYR Alexander S Azuar A Zhang J Koirala P Burne TH. Synthetic anti-cocaine nanoaccine successfully prevents cocaine-induced hyperlocomotion. J Med Chem. (2023) 66:12407–19. doi: 10.1021/acs.jmedchem.3c00889 37646732

[19] da Silva Neto L da Silva Maia AF Godin AM de Almeida Augusto PS Pereira RLG Caligiorne SM. Calix[n]arene-based immunogens: A new non-proteic strategy for anti-cocaine vaccine. J Adv Res. (2021) 38:285–98. doi: 10.1016/j.jare.2021.09.003 PMC9091763 35572397

[20] Rudra JS Ding Y Neelakantan H Ding C Appavu R Stutz S. Suppression of cocaine-evoked hyperactivity by self-adjuvanting and multivalent peptide nanofiber vaccines. ACS Chem Neurosci. (2016) 7:546–52. doi: 10.1021/acschemneuro.5b00345 PMC4871789 26926328

[21] Hicks MJ De BP Rosenberg JB Davidson JT Moreno AY Janda KD. Cocaine analog coupled to disrupted adenovirus: a vaccine strategy to evoke high-titer immunity against addictive drugs. Mol Ther. (2011) 19:612–9. doi: 10.1038/mt.2010.280 PMC3048190 21206484

[22] Ettinger RH Ettinger WF Harless WE. Active immunization with cocaine-protein conjugate attenuates cocaine effects. Pharmacol Biochem Behav. (1997) 58:215–20. doi: 10.1016/S0091-3057(97)00005-1 9264094

[23] Fox BS Kantak KM Edwards MA Black KM Bollinger BK Botka AJ. Efficacy of a therapeutic cocaine vaccine in rodent models. Nat Med. (1996) 2:1129–32. doi: 10.1038/nm1096-1129 8837612

[24] Fox BS. Development of a therapeutic vaccine for the treatment of cocaine addiction. Drug Alcohol Depend. (1997) 48:153–8. doi: 10.1016/S0376-8716(97)00121-X 9449013

[25] Carrera MR Ashley JA Zhou B Wirsching P Koob GF Janda KD. Cocaine vaccines: antibody protection against relapse in a rat model. Proc Natl Acad Sci USA. (2000) 97:6202–6. doi: 10.1073/pnas.97.11.6202 PMC18582 10823960

[26] Wee S Hicks MJ De BP Rosenberg JB Moreno AY Kaminsky SM. Novel cocaine vaccine linked to a disrupted adenovirus gene transfer vector blocks cocaine psychostimulant and reinforcing effects. Neuropsychopharmacology. (2012) 37:1083–91. doi: 10.1038/npp.2011.200 PMC3306868 21918504

[27] De BP Pagovich OE Hicks MJ Rosenberg JB Moreno AY Janda KD. Disrupted adenovirus-based vaccines against small addictive molecules circumvent anti-adenovirus immunity. Hum Gene Ther. (2013) 24:58–66. doi: 10.1089/hum.2012.163 23140508 PMC3555097

[28] Kantak KM Collins SL Lipman EG Bond J Giovanoni K Fox BS. Evaluation of anti-cocaine antibodies and a cocaine vaccine in a rat self-administration model. Psychopharmacol (Berl). (2000) 148:251–62. doi: 10.1007/s002130050049 10755738

[29] Kantak KM Collins SL Bond J Fox BS. Time course of changes in cocaine self-administration behavior in rats during immunization with the cocaine vaccine IPC-1010. Psychopharmacol (Berl). (2001) 153:334–40. doi: 10.1007/s002130000555 11271406

[30] Koob G Hicks MJ Wee S Rosenberg JB De BP Kaminsky SM. Anti-cocaine vaccine based on coupling a cocaine analog to a disrupted adenovirus. CNS Neurol Disord Drug Targets. (2011) 10:899–904. doi: 10.2174/187152711799219334 22229312 PMC3369545

[31] Evans SM Foltin RW Hicks MJ Rosenberg JB De BP Janda KD. Efficacy of an adenovirus-based anti-cocaine vaccine to reduce cocaine self-administration and reacqusition using a choice procedure in rhesus macaques. Pharmacol Biochem Behav. (2016) 150-151:76–86. doi: 10.1016/j.pbb.2016.09.008 27697554 PMC5145743

[32] Lowell JA Dikici E Joshi PM Landgraf R Lemmon VP Daunert S. Vaccination against cocaine using a modifiable dendrimer nanoparticle platform. Vaccine. (2020) 38:7989–97. doi: 10.1016/j.vaccine.2020.10.041 33158592

[33] Kimishima A Olson ME Janda KD. Investigations into the efficacy of multi-component cocaine vaccines. Bioorg Med Chem Lett. (2018) 28:2779–83. doi: 10.1016/j.bmcl.2017.12.043 PMC6013332 29317163

[34] Carrera MR Ashley JA Parsons LH Wirsching P Koob GF Janda KD. Suppression of psychoactive effects of cocaine by active immunization. Nature. (1995) 378:727–30. doi: 10.1038/378727a0 7501020

[35] Carrera MR Ashley JA Wirsching P Koob GF Janda KD. A second-generation vaccine protects against the psychoactive effects of cocaine. Proc Natl Acad Sci USA. (2001) 98:1988–92. doi: 10.1073/pnas.98.4.1988 PMC29369 11172063

[36] Cai X Whitfield T Moreno AY Grant Y Hixon MS Koob GF. Probing the effects of hapten stability on cocaine vaccine immunogenicity. Mol Pharm. (2013) 10:4176–84. doi: 10.1021/mp400214w PMC3946501 23927436

[37] Cai X Whitfield T Hixon MS Grant Y Koob GF Janda KD. Probing active cocaine vaccination performance through catalytic and noncatalytic hapten design. J Med Chem. (2013) 56:3701–9. doi: 10.1021/jm400228w PMC3691275 23627877

[38] Kosten TA Shen XY Kinsey BM Kosten TR Orson FM. Attenuation of cocaine-induced locomotor activity in male and female mice by active immunization. Am J Addict. (2014) 23:604–7. doi: 10.1111/j.1521-0391.2014.12152.x PMC4184912 25251469

[39] St John AL Choi HW Walker QD Blough B Kuhn CM Abraham SN. Novel mucosal adjuvant, mastoparan-7, improves cocaine vaccine efficacy. NPJ Vaccines. (2020) 5:12. doi: 10.1038/s41541-020-0161-1 32047657 PMC7002721

[40] Lin M Marin A Ellis B Eubanks LM Andrianov AK Janda KD. Polyphosphazene: A new adjuvant platform for cocaine vaccine development. Mol Pharm. (2022) 19:3358–66. doi: 10.1021/acs.molpharmaceut.2c00489 35984034

[41] Anton B Salazar A Flores A Matus M Marin R Hernandez JA. Vaccines against morphine/heroin and its use as effective medication for preventing relapse to opiate addictive behaviors. Hum Vaccine. (2009) 5:214–29. doi: 10.4161/hv.5.4.7556 19242094

[42] Barbosa-Méndez S Matus-Ortega M Hernandez-Miramontes R Salazar-Juárez A. Effect of the morphine/heroin vaccine on opioid and non-opioid drug-induced antinociception in mice. Eur J Pharmacol. (2021) 891:173718. doi: 10.1016/j.ejphar.2020.173718 33171151

[43] Barbosa-Mendez S Matus-Ortega M Hernandez-Miramontes R Salazar-Juárez A. M_3_-TT vaccine decrease the antinociceptive effects of morphine and heroin in mouse. Int J Ment Health Addiction. (2021) 23:783–802. doi: 10.1007/s11469-021-00621-z

[44] Salazar-Juárez A Barbosa-Méndez S Jurado N Hernández-Miramontes R Leff P Antón B. Mirtazapine prevents induction and expression of cocaine-induced behavioral sensitization in rats. Prog Neuropsychopharmacol Biol Psychiatry. (2016) 68:15–24. doi: 10.1016/j.pnpbp.2016.02.010 26922897

[45] Maoz A Hicks MJ Vallabhjosula S Synan M Kothari PJ Dyke JP. Adenovirus capsid-based anti-cocaine vaccine prevents cocaine from binding to the nonhuman primate CNS dopamine transporter. Neuropsychopharmacology. (2013) 38:2170–8. doi: 10.1038/npp.2013.114 PMC3773666 23660705

[46] Hicks MJ Kaminsky SM De BP Rosenberg JB Evans SM Foltin RW. Fate of systemically administered cocaine in nonhuman primates treated with the dAd5GNE anticocaine vaccine. Hum Gene Ther Clin Dev. (2014) 25:40–9. doi: 10.1089/humc.2013.231 PMC4047994 24649839

[47] Lockner JW Eubanks LM Choi JL Lively JM Schlosburg JE Collins KC. Flagellin as carrier and adjuvant in cocaine vaccine development. Mol Pharm. (2015) 12:653–62. doi: 10.1021/mp500520r PMC4319694 25531528

[48] Kimishima A Wenthur CJ Eubanks LM Sato S Janda KD. Cocaine vaccine development: evaluation of carrier and adjuvant combinations that activate multiple toll-like receptors. Mol Pharm. (2016) 13:3884–90. doi: 10.1021/acs.molpharmaceut.6b00682 PMC6381837 27717287

[49] Schabacker DS Kirschbaum KS Segre M. Exploring the feasibility of an anti-idiotypic cocaine vaccine: analysis of the specificity of anticocaine antibodies (Ab1) capable of inducing Ab2beta anti-idiotypic antibodies. Immunology. (2000) 100:48–56. doi: 10.1046/j.1365-2567.2000.00004.x 10809958 PMC2326984

[50] Jacob NT Anraku K Kimishima A Zhou B Collins KC Lockner JW. Bioconjugate leveraging xenoreactive antibodies to alleviate cocaine-induced behavior. Chem Commun (Camb). (2017) 53:8156–9. doi: 10.1039/c7cc04055e PMC5559285 28677711

[51] Carroll ME Zlebnik NE Anker JJ Kosten TR Orson FM Shen X. Combined cocaine hydrolase gene transfer and anti-cocaine vaccine synergistically block cocaine-induced locomotion. PloS One. (2012) 7:e43536. doi: 10.1371/journal.pone.0043536 22912888 PMC3422258

[52] Cai X Tsuchikama K Janda KD. Modulating cocaine vaccine potency through hapten fluorination. J Am Chem Soc. (2013) 135:2971–4. doi: 10.1021/ja400356g PMC3637684 23398531

[53] Steketee JD Kalivas PW. Drug wanting behavioral sensitization and relapse to drug-seeking behavior. Pharmacol Rev. (2011) 63:348–65. doi: 10.1124/pr.109.001933 PMC3082449 21490129

[54] Delage C Morel A de Witt P Jauffret-Roustide M Bloch V Noble F. Behavioral sensitization to psychostimulants and opioids: What is known in rodents and what still needs to be explored in humans? Prog Neuropsychopharmacol Biol Psychiatry. (2023) 127:110824. doi: 10.1016/j.pnpbp.2023.110824 37479108

[55] Foltin RW Fischman MW. Residual effects of repeated cocaine smoking in humans. Drug Alcohol Depend. (1997) 47:117–24. doi: 10.1016/S0376-8716(97)00093-8 9298333

[56] Ward AS Haney M Fischman MW Foltin RW. Binge cocaine selfadministration by humans: smoked cocaine. Behav Pharmacol. (1997) 8:736–44. doi: 10.1097/00008877-199712000-00009 9832960

[57] Ward AS Haney M Fischman MW Foltin RW. Binge cocaine selfadministration in humans: intravenous cocaine. Psychopharmacology. (1997) 132:375–81. doi: 10.1007/s002130050358 9298515

[58] Perrine SA Schroeder JA Unterwald EM. Behavioral sensitization to binge pattern cocaine administration is not associated with changes in protein levels of four major G-proteins. Mol Brain. Res. (2005) 133:224–32. doi: 10.1016/j.molbrainres.2004.10.025 15710239

[59] Schlussman SD Ho A Zhou Y Curtis AE Kreek MJ. Effects of “Binge” Pattern cocaine on stereotypy and locomotor activity in C57BL/6J and 129/J mice. Pharmacol Biochem Behav. (1998) 60:593–9. doi: 10.1016/S0091-3057(98)00047-1 9632245

[60] Segal DS Kuczenski R. Behavioral alterations induced by an escalating dose binge pattern of cocaine administration. Behav Brain Res. (1997) 88:251–60. doi: 10.1016/S0166-4328(97)00067-3 9404634

