# Supplementary material for: COT-TT vaccine attenuates induction and expression of cocaine-induced behavioral sensitization in rats: a dose-response study
Source: Front Psychiatry. 2025 Jun 3;16:1548585. doi: 10.3389/fpsyt.2025.1548585 (PMC12170527; doi:10.3389/fpsyt.2025.1548585)
Supplement: Supplementary file 1 [file Table1.doc]

**Supplementary Material.**

**2.3 Synthesis of the COC-TT vaccine**

The synthesis procedure of the COC-TT vaccine was divided into three stages. The first stage consists of the production of benzoylecgonine (BE). For this purpose, cocaine hydrochloride was dissolved at pH 9.5 in 0.1 M phosphate-buffered saline (PBS) at a temperature of 70 °C. Coupling of BE to water-soluble 1-(3-dimethylaminopropyl)-3-ethylcarbodiimide (EDC, Pierce, Rockford, IL, USA) was then performed.

In the second stage, the TT-TFCS conjugate tetanus toxoid+N-(ε-trifluoroacetyl caproyloxy) succinimide ester) was prepared. To do this, TFCS (Pierce, Rockford, IL, USA) was dissolved in a freshly prepared solution of 10-20% DMSO (Sigma-Aldrich, St. Louis, MO, USA)/80% distilled H2O. Subsequently, in a volume of 4 ml of PBS, pH 7.2, TFCS was mixed with tetanus toxoid (TT) and incubated at room temperature overnight. The trifluoroacetyl protecting group of TFCS, which is required to couple TFCS to the ε-amino groups of the side chain of the lysine residues of TT, was removed by incubation of the TT-TFCS solution at pH 8.1 in PBS at room temperature for up to 3 hours. Finally, the TT-TFCS derivative was purified by exhaustive dialysis against PBS, pH 7.2.

In the third stage, the COC-TT conjugate was prepared. To prepare the COC-TT conjugate, the activated EDC-COC solution was added to the TT-TFCS solution in a volume of 100 ml of PBS, pH 7.5. This reaction mixture was incubated under gentle stirring at room temperature overnight. To purify the resulting conjugate, exhaustive dialysis was performed against PBS, pH 7.4, and concentrated by pressure dialysis. The resulting solution (COC-TT) was aliquoted (unit dose = 1 mg TT/ml) and stored in sealed sterile glass vials. The COC BSA conjugate was synthesized using the same method as for TT.

**2.5 Determination of serum antibody titters via ELISA**

A solid-phase antibody capture ELISA was used to determine antibody titer responses after each booster in animals vaccinated with the COC-TT or TT vaccine. For this, ELISA plates were coated with the COC-BSA conjugate. Subsequently, a variety of serial dilutions of antisera were added in triplicate. Immunopositive signals were detected by using a biotin-labeled anti-mouse secondary antibody (Jackson Immunoresearch, West Grove, PA, USA), coupled to an OPD system (Sigma-Aldrich, St. Louis, MO, USA) as a chromogenic substrate. The reciprocal of the serum dilution that gave 50% of the maximum response was defined as the antibody titter.

| **GROUP** | **TREATMENT** | | | | |
| --- | --- | --- | --- | --- | --- |
|  | **Induction** | **Immunization** | **Expression** | **Post immunization** | **Memory** |
| **TT + SAL_-10_** | **Saline** | **Saline + TT** | **Saline** | **Saline** | **Saline + TT** |
| **TT + SAL_-40_** | **Saline** | **Saline + TT** | **Saline** | **Saline** | **Saline + TT** |
| **TT + COC_-10_** | **Cocaine_-10mg_** | **Saline + TT** | **Cocaine_-10mg_** | **Cocaine_-10mg_** | **Cocaine_-10mg_ + TT** |
| **TT + COC_-40_** | **Cocaine_-40mg_** | **Saline + TT** | **Cocaine_-40mg_** | **Cocaine_-40mg_** | **Cocaine_-40mg_ + TT** |
| **COC-TT + SAL_-10_** | **Saline** | **Saline + COC-TT** | **Saline** | **Saline** | **Saline + COC-TT** |
| **COC-TT + SAL_-40_** | **Saline** | **Saline + COC-TT** | **Saline** | **Saline** | **Saline + COC-TT** |
| **COC-TT_-20μg_ + COC_-10_** | **Cocaine_-10mg_** | **Saline + COC-TT_-20μg_** | **Cocaine_-10mg_** | **Cocaine_-10mg_** | **Cocaine_-10mg_ + COC-TT_-20μg_** |
| **COC-TT_-20μg_ + COC_-40_** | **Cocaine_-40mg_** | **Saline + COC-TT_-20μg_** | **Cocaine_-40mg_** | **Cocaine_-40mg_** | **Cocaine_-40mg_ + COC-TT_-20μg_** |
| **COC-TT_-50μg_ + COC_-10_** | **Cocaine_-10mg_** | **Saline + COC-TT_-50μg_** | **Cocaine_-10mg_** | **Cocaine_-10mg_** | **Cocaine_-10mg_ + COC-TT_-50μg_** |
| **COC-TT_-50μg_ + COC_-40_** | **Cocaine_-40mg_** | **Saline + COC-TT_-50μg_** | **Cocaine_-40mg_** | **Cocaine_-40mg_** | **Cocaine_-40mg_ + COC-TT_-50μg_** |
| **COC-TT_-100μg_ + COC_-10_** | **Cocaine_-10mg_** | **Saline + COC-TT_-100μg_** | **Cocaine_-10mg_** | **Cocaine_-10mg_** | **Cocaine_-10mg_ + COC-TT_-100μg_** |
| **COC-TT_-100μg_ + COC_-40_** | **Cocaine_-40mg_** | **Saline + COC-TT_-100μg_** | **Cocaine_-40mg_** | **Cocaine_-40mg_** | **Cocaine_-40mg_ + COC-TT_-100μg_** |

**Table I**
